# Editorial for the Special Issue “The Role of Bioactives in Inflammation, 2nd Edition”

**DOI:** 10.3390/cimb48050539

**Published:** 2026-05-21

**Authors:** Chan-Yen Kuo

**Affiliations:** Institute of Oral Medicine and Materials, College of Medicine, Tzu Chi University, Hualien 970, Taiwan; cykuo135@gms.tcu.edu.tw

Inflammation is increasingly recognized as a dynamic and interconnected regulatory network rather than a linear signaling cascade, in which immune signaling, oxidative stress, metabolic adaptation, and tissue-specific microenvironmental factors collectively shape inflammatory responses [1,2,3]. This systems-level perspective reflects a shift away from reductionist models focused on single pathways toward a more integrated framework that accounts for pathway crosstalk, cellular plasticity, and context-dependent regulation [4,5,6]. Consequently, conventional single-target anti-inflammatory strategies may not fully address the complexity and adaptive behavior of inflammatory networks.

Within this emerging framework, bioactive compounds have gained increasing attention because of their ability to modulate multiple interconnected inflammatory processes simultaneously [7]. Rather than functioning solely as pathway-specific inhibitors, these compounds often influence cytokine signaling, redox homeostasis, immune-cell activity, and tissue repair processes in a coordinated manner [8,9]. Such multi-target regulatory properties suggest that bioactive compounds may be particularly suitable for restoring inflammatory balance in complex pathological conditions.

Recent studies highlighted in this Special Issue further support the concept that bioactive compounds exert anti-inflammatory effects through integrated and context-dependent mechanisms. Exosome-associated bioactives, phytochemicals, marine-derived compounds, and multicomponent formulations have been shown to regulate inflammatory signaling, oxidative stress, epithelial integrity, and microbiota composition simultaneously [10,11,12,13,14,15,16,17]. In contrast, certain compounds, such as angelic acid, exhibit pathway-selective modulation, indicating that effective therapeutic intervention may also be achieved through precise targeting of key signaling nodes within inflammatory networks [18]. Furthermore, Kim et al. demonstrated that pyrogallol-rich rosebud extracts suppress allergic and inflammatory responses by modulating mast cell activation, oxidative stress, and pro-inflammatory mediator production, further supporting the therapeutic potential of plant-derived bioactive compounds in inflammatory disorders [19]. Collectively, these findings emphasize the growing transition from traditional single-pathway inhibition toward systems-oriented, mechanistically refined anti-inflammatory strategies.

In conclusion, the studies presented in this Special Issue collectively reinforce a paradigm shift in inflammation research from a reductionist, single-pathway perspective toward a systems-level understanding of complex, adaptive inflammatory networks. Bioactive compounds have emerged not only as isolated inhibitors but also as dynamic modulators capable of reprogramming interconnected signaling, metabolic, and immune pathways to restore tissue homeostasis. Importantly, their multi-target and context-dependent actions position them as promising candidates for addressing the intrinsic redundancy and plasticity of inflammatory processes. However, translating these insights into clinical application will require integrative approaches that combine multi-omics profiling, mechanistic validation, and well-designed clinical studies to define the efficacy, specificity, and safety of bioactive compounds. Moving forward, the convergence of systems biology and bioactive compound research is expected to broaden precision anti-inflammatory strategies and ultimately advance the development of more effective and sustainable therapeutic interventions.
